# Foot-and-Mouth Disease Virus 3C Protease Induces Fragmentation of the Golgi Compartment and Blocks Intra-Golgi Transport

**DOI:** 10.1128/JVI.01355-13

**Published:** 2013-11

**Authors:** Zhigang Zhou, Mette M. Mogensen, Penny P. Powell, Stephen Curry, Thomas Wileman

**Affiliations:** Norwich Medical School, Faculty of Medicine and Health, University of East Anglia, Norwich, United Kingdoma; Department of Cardiovascular Medicine, Medical College, Nantong University, Nantong, Chinab; Department of Biological Sciences, University of East Anglia, Norwich, United Kingdomc; Department of Life Sciences, Imperial College, London, United Kingdomd

## Abstract

Picornavirus infection can cause Golgi fragmentation and impose a block in the secretory pathway which reduces expression of major histocompatibility antigens at the plasma membrane and slows secretion of proinflammatory cytokines. In this study, we show that Golgi fragmentation and a block in secretion are induced by expression of foot-and-mouth disease virus (FMDV) 3C^pro^ and that this requires the protease activity of 3C^pro^. 3C^pro^ caused fragmentation of early, medial, and late Golgi compartments, but the most marked effect was on early Golgi compartments, indicated by redistribution of ERGIC53 and membrin. Golgi fragments were dispersed in the cytoplasm and were able to receive a model membrane protein exported from the endoplasmic reticulum (ER). Golgi fragments were, however, unable to transfer the protein to the plasma membrane, indicating a block in intra-Golgi transport. Golgi fragmentation was coincident with a loss of microtubule organization resulting from an inhibition of microtubule regrowth from the centrosome. Inhibition of microtubule regrowth also required 3C^pro^ protease activity. The loss of microtubule organization induced by 3C^pro^ caused Golgi fragmentation, but loss of microtubule organization does not block intra-Golgi transport. It is likely that the block of intra-Golgi transport is imposed by separate actions of 3C^pro^, possibly through degradation of proteins required for intra-Golgi transport.

## INTRODUCTION

The genomes of the Picornaviridae, such as poliovirus and foot-and-mouth disease virus (FMDV), synthesize a single polyprotein (P1-P2-P3) which is processed by the 3C protease (3C^pro^) at multiple specific sites to generate 11 major viral proteins (VP1 to VP4, 2A^pro^, 2B, 2C, 3A, 3B, 3C^pro^, and 3D). Processing of VP1 to VP4 proteins located at the N terminus of the polyprotein by 3C^pro^ generates the capsid proteins. The RNA-dependent RNA polymerase, 3D protein (3D^pol^), is cleaved from the C terminus, and the production of 2B, 2C, 3A, and 3B proteins and their proteolytic precursors, e.g., 2BC and 3AB, provides a scaffold for replicase assembly on cellular membranes. The 3C^pro^ of FMDV is a chymotrypsin-like cysteine protease. The active site contains a chymotrypsin-like fold and a Cys^163^-His^46^-Asp^84^ catalytic triad at the center of a peptide-binding cleft that primarily recognizes at least four residues on the N-terminal side of polyprotein cleavage junction and two on the C-terminal side (1–3). FMDV 3C has a somewhat broader specificity than other picornaviral 3C proteases; the cleavage sites contain glutamine followed by small residues (Gly, Ser, and Ala) or glutamate followed by larger, more apolar residues (Leu, Ile, and Thr) (4). The enzyme also has a patch of basic residues opposite the active site that can bind RNA and may have a role in helping to coordinate complexes formed during the initiation of viral RNA replication (5).

Infection of cells with picornaviruses results in the generation of endoplasmic reticulum (ER) and/or Golgi compartment-derived membrane vesicles that bind replicase proteins (6–8). The vesicles also contain newly synthesized viral RNA and are thought to facilitate replication and/or shield virus genomes from recognition by cellular helicases such as Rig1 and MDA5, both of which can initiate interferon responses. This remodeling of ER and Golgi membranes is coincident with a block in the secretory pathway which is thought to provide further protection from immune surveillance. For enteroviruses, the block in secretion reduces major histocompatibility complex (MHC) class I and tumor necrosis factor (TNF) receptor expression at the plasma membrane and slows secretion of interleukin-6 (IL-6), IL-8, and beta interferon (9–11). FMDV also generates membrane vesicles (12, 13) and blocks the secretory pathway (14, 15). This reduces expression of MHC class I at the plasma membrane of infected cells (16) and may reduce immune responses to FMDV and facilitate establishment of persistent infection.

The importance of the block in secretion in the context of immune evasion has led to a search for picornavirus proteins that inhibit the secretory pathway when expressed alone or in combination in cells. Up until now, these studies have focused on proteins such as 2B, 2BC, 2C, and 3A, all of which have membrane-binding motifs and associate with membrane vesicles induced during infection. Poliovirus and coxsackievirus 3A proteins slow secretion by modulating the function of Arf1 small GTPase to block ER-to-Golgi transport (17–19). The ability of 3A proteins to block secretion, however, varies among picornaviruses. The 3A proteins of human rhinovirus 14, FMDV, hepatitis A virus, or Theiler's virus do not affect secretion (20), and for FMDV, ER-to-Golgi transport is blocked by 2BC (14) or coexpression of 2B and 2C (15).

Unlike the membrane-associated proteins listed above, the picornavirus 3C^pro^ is a soluble protein located primarily in the cytoplasm. We have shown previously (21) that 3C^pro^ of FMDV causes loss of tubulin organization and disruption of the microtubule organizing center (MTOC). In this study, we show that 3C^pro^ also causes fragmentation of the Golgi apparatus and blocks the movement of proteins from the Golgi apparatus to the plasma membrane. This provides new insight into the mechanisms used by viral proteins to modulate the secretory pathway, where the protease activity of FMDV 3C^pro^, rather than membrane association, allows a viral nonstructural protein to block secretion.

## MATERIALS AND METHODS

### Plasmids.

Active FMDV 3C^pro^ mutants carrying C142S, C142A, or C142L and an inactive mutant carrying C163L (22) were generated from donor vector 3C pET28b carrying corresponding mutations. PCR-amplified 3C^pro^ products were inserted into mCherry-C1 expression vector (Clontech, CA) to make 3C^pro^ carrying an N-terminal Cherry tag. Vesicular stomatitis virus O45 temperature-sensitive glycoprotein (VSV TsO45 G) fused to yellow fluorescent protein (YFP) was described previously (14).

### Protein expression.

Vero cells (ECACC 84113001; Wiltshire, United Kingdom) were grown in 5% CO_2_ at 37°C in HEPES-buffered Dulbecco modified Eagle medium (DMEM) containing 10% fetal calf serum, 100 SI units of penicillin/ml, 100 mg/ml of streptomycin/ml, and 20 mM l-glutamine. Cells were transfected with plasmid DNA using Transfast (Promega, Southampton, United Kingdom) in DMEM for 1 h at 37°C.

### Antibodies.

Rabbit antibody β-COP was raised to the synthetic peptide CKKEAGELKPEEEITVGPVQK. A mouse monoclonal antibody (I1) recognizing the ectodomain of the VSVG protein was a gift from Douglas Lyles (Wake Forest University School of Medicine, Wake Forest, NC). Mouse anti-α- and anti-γ-tubulin and anti-membrin were purchased from Sigma (Poole, United Kingdom). Anti-GM130 was from BD Biosciences (Oxford, United Kingdom). Antibody against TGN46 was a gift from Vas Ponnambalam, University of Leeds, United Kingdom.

### Immunofluorescence microscopy.

Cells seeded on glass coverslips (Agar Scientific, Stansted, United Kingdom) were transfected *in situ* and fixed in 4% paraformaldehyde. Cells were permeabilized and blocked in 50 mM Tris (pH 7.4), 150 mM NaCl, 1% (wt/vol) gelatin, 1% (vol/vol) Nonidet P-40, 30% normal goat serum. Primary antibodies were detected with Alexa 488-, Alexa 568-, or Alexa 633-conjugated species-specific immunoglobulins (Molecular Probes through Invitrogen). DNA was stained with 50 ng/ml DAPI (4′,6-diamidino-2-phenylindole). Coverslips were mounted in Vectashield (Vector Laboratories, Peterborough, United Kingdom).

### Microtubule regrowth.

Cells grown on coverslips expressing FMDV 3C^pro^ fused to mCherry were incubated with 2.5 μM nocodazole for 1 h in ice followed by an additional 1 h at 37°C. Cells were washed twice in ice-cold phosphate-buffered saline and incubated in cell culture medium at 37°C for 5 min to allow microtubule regrowth. Samples were fixed in methanol (−20°C) at increasing times and immunostained for α-tubulin.

## RESULTS

### FMDV 3C^pro^ causes Golgi fragmentation.

**Fig 1:**
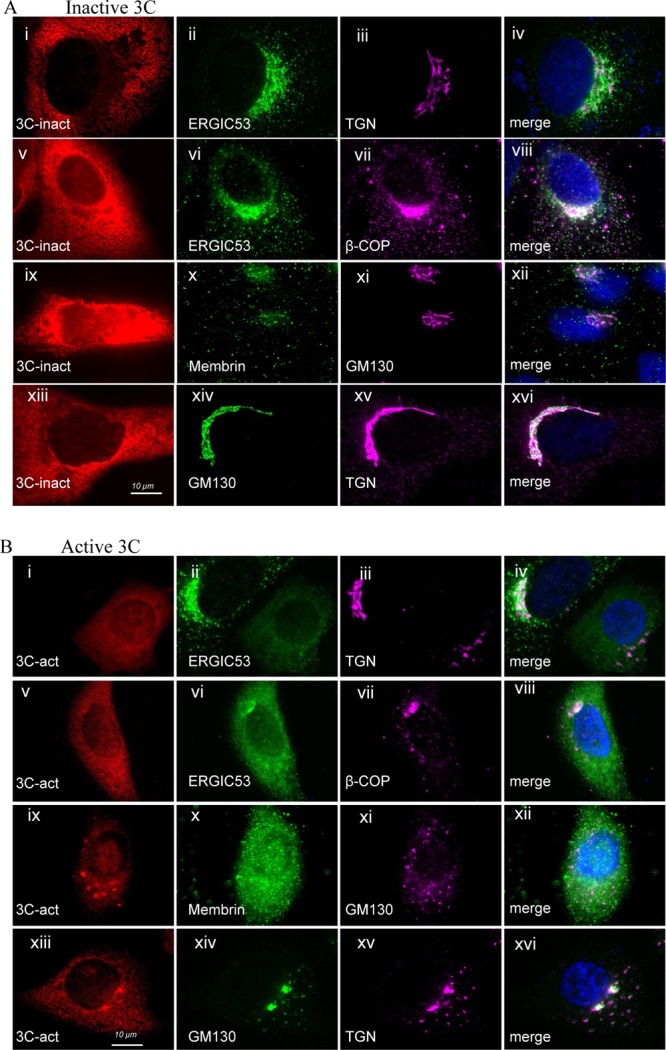
The protease activity of FMDV 3C^pro^ is required to induce Golgi fragmentation. Vero cells expressing inactive FMDV 3C^pro^ (A) or active 3C^pro^ (B) fused to mCherry (red) were fixed, permeabilized, and immunostained for ERGIC53, membrin, β-COP, GM130, and TGN46 as indicated. Nuclei were visualized with DAPI (blue). Merged images compare signals from Golgi markers.

**Fig 2:**
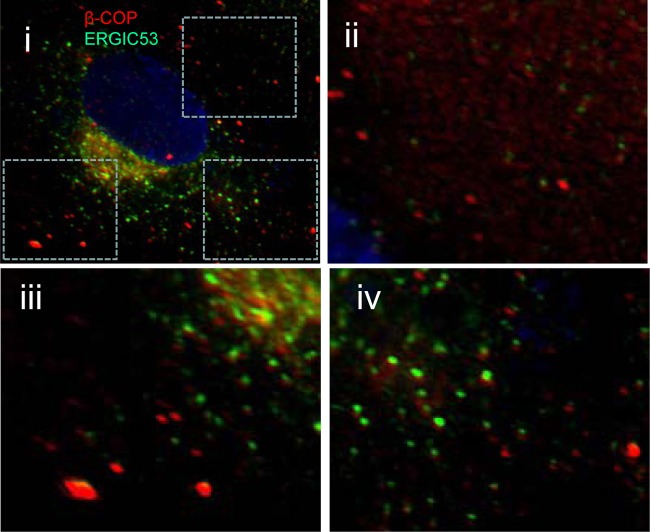
ERGIC53 and β-COP do not colocalize. Vero cells were fixed, permeabilized, and immunostained for ERGIC53 (green) and β-COP (red). Nuclei were visualized with DAPI (blue). Panel i shows a merged image. Regions of interest taken from the merged image (boxed) are presented at higher magnification (ii to iv) to show separate signals for ERGIC53 and β-COP.

Disruption of microtubule organization, for example, by depolymerizing microtubules with nocodazole, results in fragmentation of the Golgi compartment into vesicles dispersed throughout the cytoplasm (23). The observation that 3C^pro^ disrupted microtubule organization (21) prompted us to test whether 3C^pro^ may also disrupt the Golgi compartment and whether this required the protease activity of the enzyme. The effect of an inactive form of 3C^pro^ on the Golgi compartment was tested by expression of an enzyme where cysteine 163 in the active site had been converted to alanine (Fig. 1A). Cells were counterstained with antibodies against early (ERGIC53 and membrin), central (β-COP and GM130), and late (TGN46) Golgi marker proteins. In the presence of inactive 3C protease (Fig. 1A, i), ERGIC53 was distributed within a series of vesicles mostly localized to one side of the nucleus (Fig. 1A, ii), and a similar distribution was seen for β-COP (Fig. 1A, vii). An analysis of vesicles in the peripheral cytoplasm showed that signals for ERGIC53 and β-COP were largely separate (Fig. 1A, viii, and Fig. 2). The white signal in the merge image resulted from the high density of vesicles containing β-COP and ERGIC53 next to the nucleus. Vesicles positive for ERGIC53 were also interspersed between but separate from vesicles and stacks containing TGN36 (Fig. 1A, iii and iv). The ER-Golgi SNARE protein membrin (Fig. 1A, x) localized in vesicles throughout the cytoplasm, and some colocalized with central Golgi marker GM130 (Fig. 1A, xi and xii). Golgi stacks remained intact in the presence of inactive 3C^pro^ indicated by the crescent of GM130 (Fig. 1A, xiv) and TGN36 (Fig. 1A, iii and xv) immunostaining next to the nucleus.

Expression of active 3C^pro^ resulted in fragmentation of all Golgi compartments (Fig. 1B), but the most marked effect was on ERGIC53 (Fig. 1B, vi) and membrin (Fig. 1B, x) distribution, leading to diffuse rather than punctate staining and ERGIC53 no longer being concentrated next to the nucleus (Fig. 1B, ii and vi). Fragmentation of the Golgi compartment by active 3C^pro^ was indicated by loss of organized perinuclear stacks and the generation of scattered vesicles containing β-COP, GM130, and TGN46 (see Fig. 1B, vii, xi, and xv, respectively). The white signal in the merged image (Fig. 1A, viii) resulted from the high density of vesicles containing β-COP and ERGIC53 next to the nucleus. An analysis of vesicles in the peripheral cytoplasm showed that signals for ERGIC53 and β-COP were largely separate (Fig. 2).

### The protease activity of FMDV 3C is required for disruption of tubulin organization.

**Fig 3:**
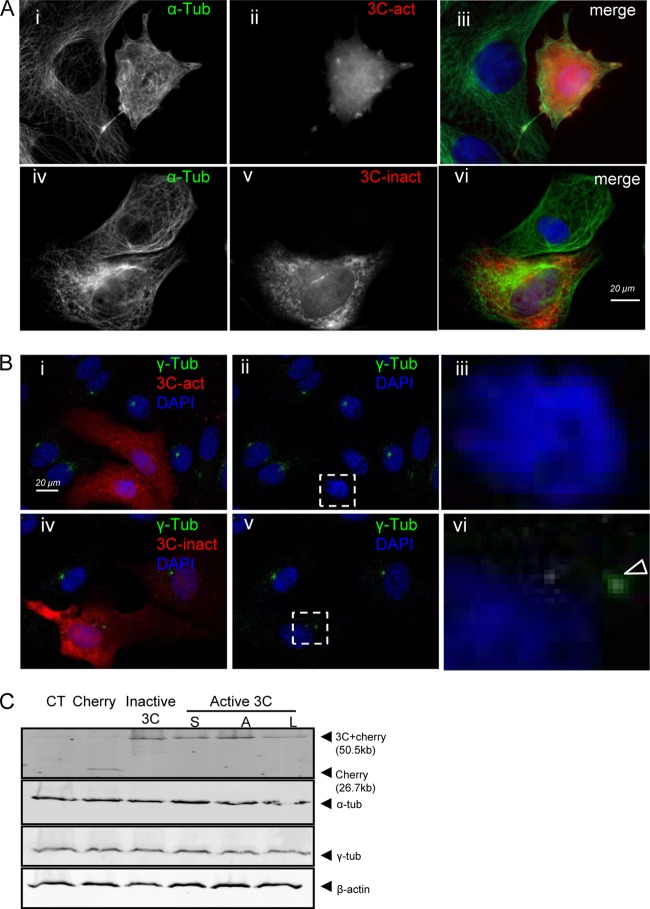
The protease activity of FMDV 3C^pro^ is required for disruption of microtubule organization. (A) Vero cells expressing active FMDV 3C^pro^ (i to iii) or inactive 3C^pro^ (iv to vi) fused to mCherry (red) were fixed, permeabilized, and immunostained for α-tubulin (green). (B) Vero cells expressing active FMDV 3C^pro^ (i to iii) or inactive 3C^pro^ (iv to vi) fused to mCherry (red) were fixed, permeabilized, and immunostained for γ-tubulin (green). Nuclei were visualized with DAPI (blue). Cells expressing 3C^pro^ are indicated with dashed boxes and shown at high magnification (iii and vi). The arrow in panel B, vi, indicates MTOC. Nuclei were visualized with DAPI (blue). (C) Control Vero cells (CT) or Vero cells expressing active FMDV 3C^pro^ or inactive 3C^pro^ fused to mCherry were lysed. Total cell lysates were analyzed by Western blotting to detect Cherry tag, α-tubulin, γ-tubulin, and β-actin.

Given that the protease activity of 3C^pro^ was required for Golgi fragmentation and that this may be related to loss of microtubules, we compared the effects of wild-type 3C^pro^ and the inactive protease on tubulin organization (Fig. 3A). Cells negative for 3C^pro^ (Fig. 3A, i and ii) spread across the coverslip and had clear internal organization of α-tubulin into microtubules distributed radially from the MTOC throughout the cytoplasm. In contrast, cells expressing 3C^pro^ showed evidence of rounding and loss of radial microtubule organization, as indicated by diffuse cytoplasmic α-tubulin staining (Fig. 3A, i). These changes did not occur in cells expressing the inactive 3C^pro^ (Fig. 3A, iv to vi). These cells remained flat, and microtubule arrays were evident throughout the cell. Loss of tubulin organization in cells expressing 3C^pro^ is coincident with loss of γ-tubulin from the MTOC (21). The role of the protease activity of 3C in dispersal of γ-tubulin was tested by comparing the distributions of γ-tubulin in cells expressing active or inactive 3C^pro^ (Fig. 3B). The punctate γ-tubulin staining close to the nucleus indicating the MTOC was absent from cells expressing the active protease (Fig. 3B, ii), but the MTOC was present in cells in the same preparation that were negative for 3C^pro^. When the experiment was repeated for inactive 3C^pro^ (Fig. 3B, iv to vi), the γ-tubulin stain was concentrated at the MTOC (Fig. 3B, v). Taken together, the results show that the protease activity of 3C is required for loss of γ-tubulin from the MTOC. This raised the possibility that γ-tubulin might be degraded by 3C^pro^. Levels of γ-tubulin, α-tubulin, and actin were therefore compared by Western blotting of cells expressing the inactive C163L 3C^pro^ and active C142S, C142A, and C142L enzymes (Fig. 3C). The signals for all three components of the cytoskeleton were the same as those observed for control cells expressing mCherry, making it likely that the loss of γ-tubulin from the MTOC resulted from dispersal into the cytoplasm, rather than from direct degradation by 3C^pro^.

### FMDV 3C^pro^ slows microtubule nucleation at the MTOC.

**Fig 4:**
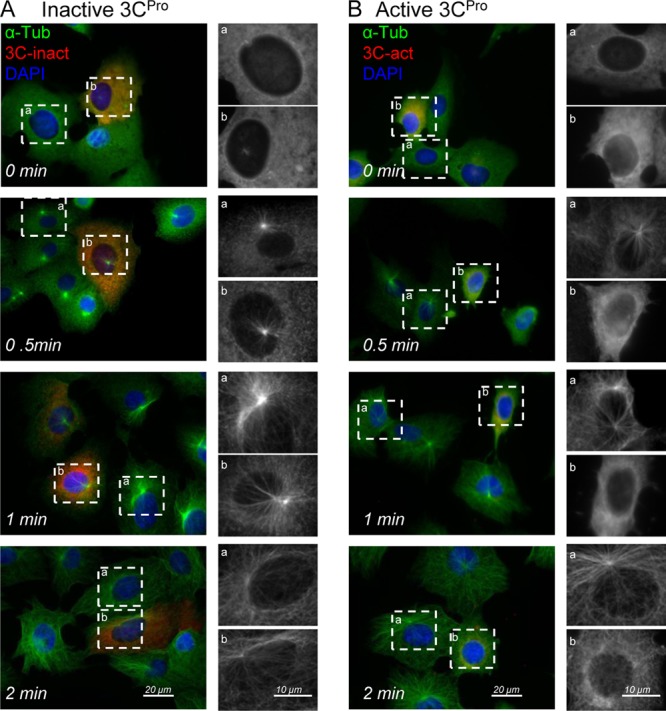
The protease activity of FMDV 3C^pro^ is required for inhibition of microtubule regrowth. Vero cells expressing inactive FMDV 3C^pro^ (A) or active 3C^pro^ (B) fused to mCherry (red) were incubated with nocodazole to depolymerize microtubules. Cells were washed and incubated in the absence of nocodazole to initiate microtubule regrowth. Cells were fixed, permeabilized, and immunostained for α-tubulin (green) at the indicated times. The regions of interest of selected 3C-positive (a) and 3C-negative (b) cells at each time point are shown at high magnification on the right of each panel (gray) to show microtubule asters at MTOC.

The loss of microtubule organization seen in cells expressing active 3C^pro^ may have occurred because centrosomes with reduced levels of γ-tubulin have reduced capacity to nucleate microtubules. Given that an ability to nucleate microtubules is also important for maintaining the structure of the Golgi compartment (23–25), the effect of 3C^pro^ on microtubule nucleation was tested using microtubule regrowth assays (Fig. 4). Microtubules were depolymerized by adding nocodazole in chilled media. Cells were washed and warmed to 37°C, and microtubules were allowed to repolymerize for increasing times prior to fixation and analysis by immunostaining for α-tubulin. Figure 4 compares microtubule regrowth in cells expressing inactive (Fig. 4A) or active (Fig. 4B) 3C^pro^. In each experiment, the boxed regions of interest compare cells expressing 3C^pro^, indicated by the mCherry signal (a), with neighboring cells negative for 3C^pro^ (b). The images taken at 0 min show that α-tubulin staining was dispersed throughout the cytoplasm following incubation with nocodazole and that there was a loss of organization of α-tubulin at the MTOC. As early as 30 s after removal of nocodazole, asters of α-tubulin staining were visible in cells lacking 3C^pro^, or expressing the inactive protease, and radial arrays of microtubules were evident at 1 min (Fig. 4A). In contrast, cells expressing the active 3C^pro^ (Fig. 4B, b) were unable to form asters of α-tubulin or microtubule arrays over the same time course, even though microtubule regrowth occurred in neighboring cells negative for mCherry 3C^pro^ signal (Fig. 4B, a). Taken together, the results show that the proteolytic activity of 3C^pro^ is required for loss of γ-tubulin from the MTOC and consequent loss of capacity to nucleate microtubules.

### 3C^pro^ slows intra-Golgi transport.

**Fig 5:**
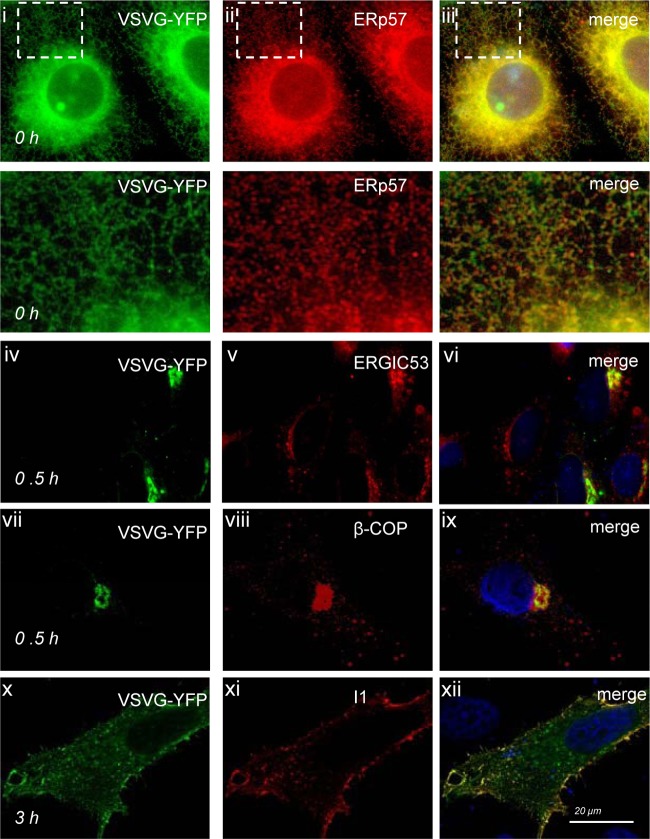
Transport of VSV TsO45 G protein from the endoplasmic reticulum to the plasma membrane. Vero cells expressing VSV TsO45 G protein fused to YFP (green) were incubated at 40°C for 40 h and then cooled to 32°C for the indicated times (hours). Cells were fixed, permeabilized, and immunostained for endoplasmic reticulum (ERp57, red, i to iii), ERGIC (ERGIC53, red, iv to vi), or Golgi compartment (β-COP, red, vii to ix). Delivery of the G protein to the plasma membrane was assessed by incubating cells with G-protein-specific antibody I1 (red) before permeabilization (x to xii). Nuclei were labeled with DAPI (blue). Merged images compare G-YFP signals with cellular markers.

The ability of Golgi fragments generated by 3C^pro^ to transport proteins was tested by following the transport of a model membrane protein, the G glycoprotein of vesicular stomatitis virus (VSVG), from the endoplasmic reticulum (ER) to the Golgi compartment and plasma membrane in cells expressing 3C^pro^. The glycoprotein of the temperature-sensitive O45 mutant of VSV (TsO45 G) is retained in the ER at 40°C but moves to the Golgi apparatus and plasma membrane when cells are cooled to 32°C. A temperature shift experiment is illustrated in Fig. 5. TsO45 G fused to YFP localized to reticular structures at 40°C which colocalized with luminal ER protein ERp57 (Fig. 5, i to iii), indicating retention in the ER. When cells were cooled to 32°C for 30 min, the TsO45 protein was localized in a perinuclear structure that codistributed with ERGIC53 (Fig. 5, iv to vi) and β-COP (Fig. 5, vii to ix), indicating movement to early Golgi compartments. The images show separate cell populations which have been analyzed with antibodies specific for ERGIC53 or β-COP. A survey of cells showed that the TsO45 G protein was localized to both vesicle populations at 30 min, indicating transit through early Golgi compartments. The results do not, however, indicate that ERGIC53 and β-COP are colocalized in these cells. An antibody specific for the ectodomain of the G protein (I1) was added to cells before permeabilization 3 h after the temperature shift to 32°C. The signal from I1 colocalized with the YFP signal (Fig. 5B, x to xii) at the perimeter of the cell, indicating delivery of TsO45 G protein to the plasma membrane.

**Fig 6:**
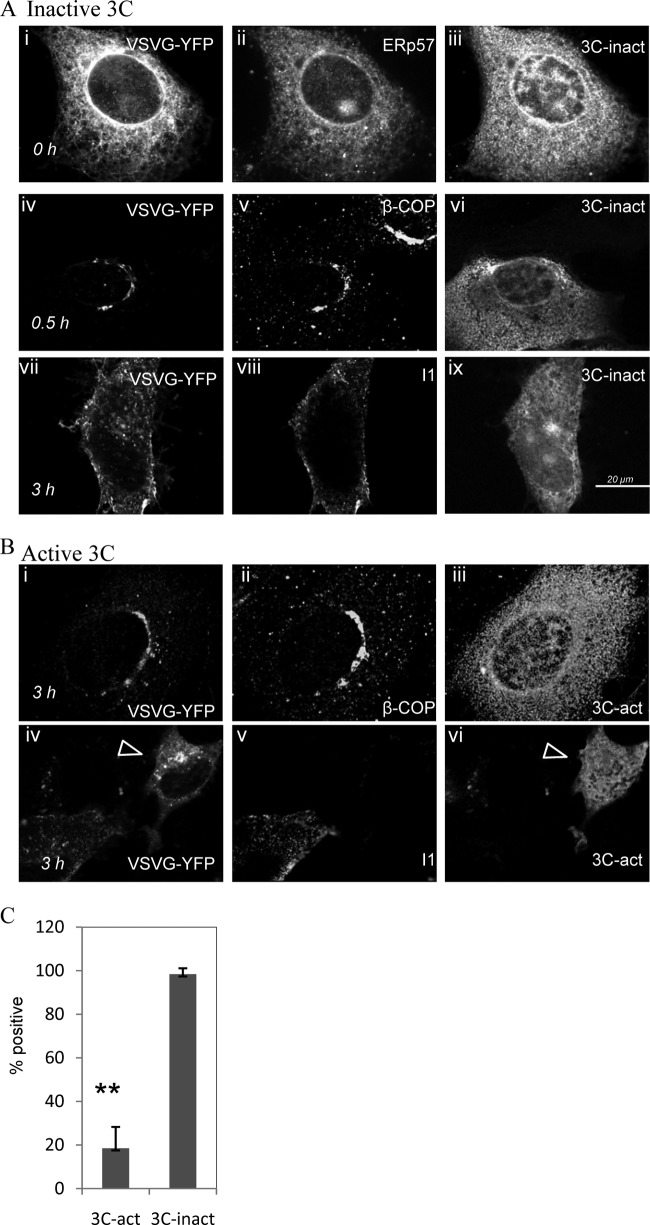
The protease activity of FMDV 3C^pro^ is required for inhibition of intra-Golgi transport. Vero cells coexpressing VSV TsO45 G protein fused to YFP and 3C^pro^ were incubated at 40°C for 40 h and cooled to 32°C for the indicated times (0, 0.5, and 3 h). (A) Cells expressing VSV TsO45 G-YFP (i, iv, and vii) and inactive 3C^pro^ fused to mCherry (iii, vi, and ix) were immunostained for endoplasmic reticulum (ERp57, ii) or Golgi compartment (β-COP, v). Delivery of the G protein to the plasma membrane was assessed by adding I1 antibody before permeabilization (I1, viii). (B) Cells expressing VSV TsO45 G-YFP (i and iv) and active 3C^pro^ fused to mCherry (iii and vi) were immunostained at 3 h for Golgi compartment (β-COP, ii). Delivery of the G protein to the plasma membrane at 3 h was assessed by adding I1 antibody before permeabilization (I1, v). The arrowhead shows a cell expressing active 3C^pro^ (vi) and VSVG-YFP (iv) which lacks I1 staining (v) at 3 h.

**Fig 7:**
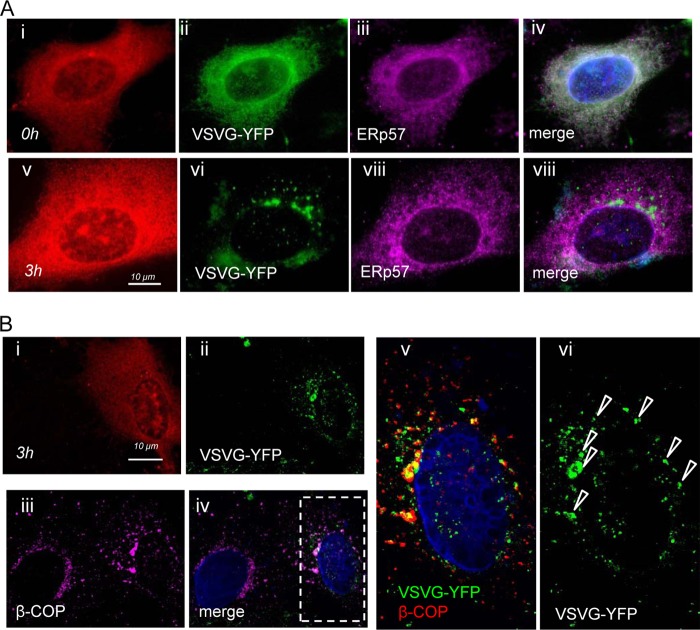
The VSVG protein is retained in the Golgi compartment in cells expressing FMDV 3C^pro^. Vero cells coexpressing VSV TsO45 G-YFP and active 3C^pro^ were incubated at 40°C for 40 h and cooled to 32°C for 3 h. (A) Cells were stained for endoplasmic reticulum (ERp57) at 0 h (i to iv) and 3 h (v to viii). (B) Cells were stained at 3 h for Golgi compartment (β-COP, iii). High magnifications of the boxed region of interest (v and vi) indicate colocalization of G protein and β-COP (arrowheads). Bar, 10 μm.

Figure 6 compares the effects of the inactive and active forms of 3C^pro^ on movement of the TsO45 G protein to the plasma membrane. Each image shows the location of TsO45 G protein in a cell expressing Cherry-tagged 3C^pro^. The inactive 3C^pro^ (Fig. 6A) had little effect on secretion. The TsO45 G protein colocalized with ERp57 at 40°C (Fig. 6A, i to iii) and with β-COP after 30 min at 32°C (Fig. 6A, iv to vi). TsO45 G protein was detected at the plasma membrane (Fig. 6A, vii to ix) after 3 h of temperature shift. In contrast, expression of the active 3C^pro^ (Fig. 6B) prevented movement of the TsO45 G protein to the plasma membrane. TsO45 G colocalized with Golgi protein β-COP at 3 h (Fig. 6B, i to iii) and was not detected at the plasma membrane. Panels iv to vi of Fig. 6B show two cells expressing VSVG TsO45-YFP after 3 h at 32°C. The G protein was detected by I1 ectodomain antibody in the plasma membrane of the cell lacking expression of active 3C^pro^, but I1 staining was absent from the expressing cell (arrowhead). The results show that the G protein is retained in the Golgi apparatus of cells expressing active 3C^pro^ and that the protease therefore slows intra-Golgi transport. The experiment was repeated three times, and cells expressing TsO45 G-YFP and 3C^pro^ were scored for delivery of TsO45 G to the plasma membrane within 3 h of temperature shift (Fig. 6C). TsO45 G was detected at the plasma membrane of 98% of cells expressing inactive 3C^pro^ but only 18% of cells expressing active enzyme. The transport of the TsO45 G protein in cells expressing active 3C^pro^ is shown in more detail in Fig. 7, where cells have been counterstained for ERp57 and β-COP after 3 h at 32°C. The TsO45 G protein colocalized with ERp57 before temperature shift (Fig. 7A, i to iv) and was separate from ERp57 after 3 h (Fig. 7A, v to viii), indicating transport from the ER. The TsO45 G protein was localized to vesicular structures after 3 h at 32°C (Fig. 7B, ii), and there was no evidence for transport to the plasma membrane. The vesicles containing TsO45 G protein colocalized with β-COP (Fig. 7B, iii to vi), indicating retention in the Golgi apparatus. The degree of colocalization was further assessed by converting the magenta signal for β-COP to red and analyzing merged images at higher magnification (Fig. 7B, v and vi). Vesicles containing VSVG-YFP (Fig. 7B, vi) appeared orange in the merged image (arrowheads in Fig. 7B, v).

## DISCUSSION

Infection of cells with picornaviruses such as FMDV and poliovirus leads to Golgi fragmentation and a block in protein secretion (10, 14, 15, 18–20). The search for viral proteins that might be responsible has concentrated on nonstructural proteins, such as 2B, 2C, 2BC, and 3A, that have membrane-targeting sequences and associate with membrane vesicles induced during viral infection. FMDV 3C^pro^ is a soluble protein found primarily in the cytoplasm of cells. Here, we show that the Golgi fragmentation and block in secretion seen during FMDV infection can be reproduced by expression of FMDV 3C^pro^. We have shown previously that infection of cells with FMDV or expression of FMDV 3C^pro^ alone causes loss of γ-tubulin from the MTOC and overall loss of microtubule organization (21). Here, we show that loss of γ-tubulin from the MTOC reduces the capacity of centrosomes to nucleate microtubules and results in fragmentation of the Golgi compartment and loss of trans-Golgi network (TGN) stacks. We also show that loss of microtubule organization and Golgi fragmentation both require the protease activity of 3C^pro^.

Early studies have shown that microtubule depolymerization leads to Golgi fragmentation (23, 24); however, microtubule depolymerization does not always lead to a block in secretion (24, 25). It is not therefore possible to conclude that Golgi fragmentation induced by the loss of microtubule organization leads to the block of intra-Golgi transport induced by FMDV 3C^pro^. When Golgi stacks are dispersed by nocodazole, secretion resumes following redistribution of Golgi membrane components to peripheral ER exit sites where they generate small Golgi “ministacks” (23). Our results show that the Golgi fragments induced by FMDV 3C^pro^ received VSVG protein exported from the ER, but unlike Golgi ministacks induced by nocodazole, the VSVG protein was trapped in Golgi fragments. We believe that the loss of microtubule organization induced by 3C^pro^ leads to Golgi fragmentation but that the block in intra-Golgi transport is imposed by separate actions of 3C^pro^.

Golgi fragmentation occurs during poliovirus infection (26). However, enteroviruses such as poliovirus and bovine enterovirus do not cause loss of microtubule organization (21, 26). For poliovirus, Golgi fragmentation may be induced by nonstructural proteins, e.g., 3A, 2B, and 2BC, that have membrane-binding domains and associate with ER and Golgi membrane compartments. The Golgi fragmentation seen during poliovirus infection begins with the formation of large heterogeneous vesicles and tubes which disperse further to form small Golgi “puncta” (22). These puncta contain Golgi markers GM130 and β-COP and, in common with Golgi fragments generated by FMDV 3C^pro^, receive secretory flow from the ER but fail to deliver VSVG to the plasma membrane. The Golgi puncta induced by poliovirus are smaller than Golgi ministacks formed following incubation with nocodazole, and the conversion of the larger fragments to Golgi puncta is inhibited by nocodazole, suggesting that late stages of fragmentation require microtubules. The Golgi fragments generated by FMDV remained large, and it is possible that their conversion to the small Golgi puncta seen during poliovirus infection is prevented during FMDV infection because 3C^pro^ disrupts microtubule organization.

It is apparent that picornaviruses can block ER-to-Golgi transport and intra-Golgi transport and that different mechanisms are employed depending on the site of the block in secretion and the particular virus under study. ER-to-Golgi transport is blocked by enterovirus 3A proteins which disrupt the function of the Arf1 GTPase. Activation of Arf1 by its guanine nucleotide exchange factor, GBF1, recruits β-COP to the Golgi compartment to initiate formation of COP1-coated transport vesicles. Binding of enterovirus 3A to GBF1 results in recruitment of an alternative Arf1 effector protein, the lipid kinase PI4KIIIβ (19). The kinase generates PI4-P to facilitate recruitment of 3D^pol^ to initiate assembly of the replicase, but preferential recruitment of PI4KIIIβ over β-COP leads to loss of COP1 coats from the Golgi compartment. Work by Beske et al. (26) suggests that a similar mechanism does not cause the block in intra-Golgi transport seen during poliovirus infection because Golgi fragments retain their COP1 coats, indicating recruitment of β-COP by Arf1, but are still defective in intra-Golgi transport.

Unlike poliovirus, the 3A proteins of many picornaviruses, e.g., human rhinovirus 14, FMDV, enterovirus 71, hepatitis A virus, and Theiler's virus, do not affect Golgi transport (14, 20). The 3A protein of human rhinovirus 1A binds to the Golgi compartment and causes Golgi fragmentation, but effects on intra-Golgi transport are not known (22). In the case of FMDV, ER-to-Golgi transport is blocked by 2BC, while coexpression of 2B with 2C blocks intra-Golgi transport (14, 15). Taken with our current study, it is clear that 3C^pro^ can provide FMDV with at least two independent means of blocking secretion, and both are dependent on protease activity. The first results when 3C^pro^ processes 2BC to generate 2B and 2C that bind the Golgi compartment and block intra-Golgi transport. The second allows 3C^pro^ to block secretion without physically binding Golgi membranes, possibly by degrading proteins required for intra-Golgi transport.
